# Hippocampal temporal dynamics and spatial heterogeneity unveil vulnerability markers in the offspring of bipolar patients

**DOI:** 10.1111/bdi.13487

**Published:** 2024-08-12

**Authors:** Luigi F. Saccaro, Farnaz Delavari, Dimitri Van De Ville, Camille Piguet

**Affiliations:** ^1^ Psychiatry Department, Faculty of Medicine University of Geneva Geneva Switzerland; ^2^ Psychiatry Department Geneva University Hospital Geneva Switzerland; ^3^ Department of Radiology and Medical Informatics University of Geneva Geneva Switzerland; ^4^ Developmental Imaging and Psychopathology Laboratory University of Geneva School of Medicine Geneva Switzerland; ^5^ Neuro‐X Institute, School of Engineering Ecole Polytechnique Fédérale de Lausanne (EPFL) Geneva Switzerland; ^6^ Child and Adolescence Psychiatry Division Geneva University Hospital Geneva Switzerland

**Keywords:** biomarkers, bipolar disorder, high‐risk, hippocampus, imaging, offspring

## Abstract

**Objectives:**

Bipolar disorder (BD) is a highly heritable disorder characterized by emotion dysregulation and recurrent oscillations between mood states. Despite the proven efficacy of early interventions, vulnerability markers in high‐risk individuals are still lacking. BD patients present structural alterations of the hippocampus, a pivotal hub of emotion regulation networks composed of multiple subregions with different projections. However, the hippocampal dynamic functional connectivity (dFC) in BD remains unclear. We aim to investigate whether the dFC of hippocampal subdivisions differentiates BD patients, offspring of BD patients (BDoff), and healthy controls (HC); and whether it correlates with symptoms differently between these groups.

**Methods:**

We studied for the first time the dFC of the hippocampus through a cutting‐edge micro‐co‐activation patterns (μCAPs) analysis of resting‐state functional MRI data of 97 subjects (26 BD, 18 BDoff, 53 HC). μCAPs allow a data‐driven differentiation within the seed region.

**Results:**

dFC between the hippocampal body and a somatomotor‐μCAP was lower both in BD patients (*p*‐value_FDR_:0.00015) and in BDoff (*p*‐value_FDR_:0.020) than in HC. Inversely, dFC between the hippocampal head and a limbic‐μCAP was higher in BD patients than in HC (*p*‐value_FDR_: 0.005). Furthermore, the correlations between a frontoparietal‐μCAP and both depression and emotion dysregulation symptoms were significantly higher in BD than HC (*p*‐value_FDR_ <0.02).

**Conclusion:**

Overall, we observed alterations of large‐scale functional brain networks associated with decreased cognitive control flexibility and disrupted somatomotor, saliency, and emotion processing in BD. Interestingly, BDoff presented an intermediate phenotype between BD and HC, suggesting that dFC of hippocampal subregions might represent a marker of vulnerability to BD.

## 1 INTRODUCTION

Bipolar disorder (BD) is a severe, chronic, affective psychiatric condition,^1^ and a significant public health concern, affecting up to 2.4% of people worldwide^1^ and being associated with significant functional impairment, decreased quality of life, and increased risk of suicide.^1^ BD is characterized by recurrent episodes of mania, depression, and mixed states, each of which has its own set of psychomotor, cognitive, and affective symptoms.^1^ Even in the euthymic state, there is often a persistence of symptoms. These so‐called residual symptoms may include for instance emotion dysregulation which may be expressed through anxio‐depressive symptoms and self‐referential negative thoughts, psychomotor alterations, or cognitive impairment.^2^

Evidence from twin studies points to an overall BD heritability of more than 70%.^3^ Despite the significant burden of BD and the importance of early interventions in alleviating it, a lack of comprehensive understanding of BD pathophysiology and neurobiological markers makes it difficult to identify effective interventions as well as vulnerability traits that may be the ideal targets for such interventions in high‐risk individuals. From a clinical perspective, the characterization of offspring of BD patients (BDoff) is thus of critical importance in order to identify potential early warning signs or vulnerability markers of BD and to guide early interventions. These interventions can help prevent or mitigate BD development, to improve the long‐term outcomes in high‐risk populations, such as Bdoff.

Alterations in several brain structures, particularly in emotion regulation networks, have been consistently associated with BD and may represent a clinically relevant vulnerability marker, thanks to the noninvasive nature of neuroimaging studies, and to the existence of multiple promising, noninvasive early interventions targeting emotion dysregulation.^4^ The hippocampus, in particular, is a brain region composed of multiple parcels with different projections, which plays a key role in cognition, memory, somatomotor (or sensorimotor) integration,^5^ and, most importantly, in emotion regulation networks, for example in the prefrontal cortical‐hippocampal‐amygdala emotion‐processing circuit.^6^ Indeed, extensive research in clinical neuroscience supports the pivotal role of the hippocampus in the pathophysiology of BD.^7^, ^8^ Interestingly, some effective pharmacological treatments for BD seem to directly influence hippocampal physiology and function,^9^ and the hippocampus is known to play a significant role in regulating the hypothalamic–pituitary–adrenal axis, a key component of the stress response system that has been found to be dysregulated in BD.^10^ While various hippocampal parcellations have been proposed, a general consensus exists on a head‐body‐tail hippocampal subdivision.^11^, ^12^ Through behavioral and functional profiling, an “emotion‐cognition gradient” along the anterior–posterior axis of the hippocampus has been identified.^12^, ^13^ Consequently, the hippocampus serves as a nexus for cognitive representations and functions (processed more posteriorly), integrating external somatomotor information with internal affective features (more anteriorly, e.g., the hippocampal head).^12^ Importantly, these functions overlap with primary domains affected by BD symptoms, namely cognition, emotion, stress regulation, and somatomotor processing.^6^, ^14^

In spite of the extensive evidence linking the hippocampus to BD, there remains a significant gap in our understanding of the underlying mechanisms and the precise role that the hippocampus plays in the vulnerability to BD, and in the residual symptoms in euthymic BD patients.

On one hand, the hippocampus emerges as a promising region for understanding BD vulnerability, given its connections to stress regulation and inflammation,^15^, ^16^ both recognized as associated with BD and potential risk factors in individuals at high risk for BD.^10^, ^17^, ^18^ Hence, hypothetically, the fact that the hippocampus is affected by these variables that constitute risk factors for BD underscores the relevance of investigating the hippocampus when studying BD vulnerability, besides its aforementioned roles in cognition, emotion, stress regulation, and somatomotor processing.^6^, ^14^

On the other hand, little is known about the relationship between the hippocampal FC and BD symptoms, and, to the best of our knowledge, just one study investigated, only in BDoff, the potential of hippocampal stationary FC in predicting the progression to full‐blown BD, yielding promising results.^19^ It is worth noting that recent methodological advancements have contributed to our comprehension of how BD impacts brain FC. Previous research on BD patients has consistently revealed disruptions in both inter‐ and intra‐network stationary functional connectivity (FC) within various large‐scale brain networks, such as the frontoparietal network (FPN), the somatomotor network (SMN), the default mode network (DMN), and the salience network (SN). Crucially, it has been proposed that abnormal stationary FC may be linked to specific BD symptoms.^20^, ^21^ For instance, in a study involving 18 BD patients, SMN FC was found to be positively correlated with the severity of manic symptoms.^22^ Interestingly, recent research has increasingly implicated the SMN in emotional and cognitive processing, making SMN a pivotal area of investigation in the study of BD^21^, ^23^, ^24^, ^25^, ^26^, ^27^ and of emotion dysregulation.^28^

Overcoming some limitations of traditional stationary FC analyses, efforts to characterize the time‐varying aspects of blood‐oxygen‐level‐dependent (BOLD) signals using dynamic FC (dFC)^29^, ^30^ have provided valuable insights into the dynamic behavior of large‐scale networks that are typically impaired in BD. However, only one study has thus far examined hippocampal dFC in relation to BD patients' symptoms.^31^ Given the heterogeneous structural and functional composition of the hippocampus, along with its intricate network of multiple projections to various brain areas, it is crucial to acknowledge that valuable information may be overlooked if the hippocampus is treated as a homogeneous region when employed as a seed in dFC studies. More studies are needed to fill these existing gaps in the literature to improve the prognosis for people living with BD and for those at high risk for the disorders.

Therefore, the present study tackles these challenges and offers novel insights on hippocampal resting‐state dFC in BD by implementing micro‐co‐activation patterns (μCAPs) analysis,^32^ a recent technique that allows for a fine‐grained, data‐driven differentiation within the seed region of interest. Unlike stationary FC analyses and building on the well‐validated CAPs technique (see^29^, ^30^ for a full description of the CAPs method), μCAPs capture the dynamic features of time‐varying brain activity, disentangling interactions between weighted contributions within the hippocampus and distinct whole‐brain patterns that include brain networks, like the FPN, SMN, and DMN. This feature becomes particularly advantageous when investigating large seed regions that participate in various brain networks associated with multiple distinct symptom domains, such as the hippocampus, which is involved in cognition, stress regulation, emotion, and somatomotor processing in BD, as detailed above. Additionally,^29^, ^30^ the data‐driven differentiation within the seed region implemented in μCAPs is particularly suited for analysis of a brain structure composed of multiple subdivisions with specialized functions and cytoarchitecture, such as the hippocampus.^12^, ^13^

Thus, building on the hypothesis that disruption of hippocampal subregions dFC may provide relevant markers of BD pathophysiology and vulnerability in Bdoff, here we aim at investigating whether (1) hippocampal dFC differentiates BD patients, BDoff, and healthy controls (HC); (2) hippocampal dFC correlates with symptoms; (3) these correlations differ between BD, BDoff, and HC. Indeed, comparing these correlations across the three groups may shed light on group‐specific patterns of dFC related to BD and to vulnerability to BD, paving the way to more targeted and effective interventions for early prevention or mitigation of BD burden in high‐risk subjects, or for managing the residual symptoms experienced by individuals with BD.

## 2 METHODS AND MATERIALS

### 2.1 Participants

A total of 97 subjects were recruited in total. Among them, 26 individuals with euthymic BD, who met the DSM‐IV‐TR criteria, were recruited from the Mood Clinic within the Psychiatry Department of Geneva University Hospitals, according to a protocol previously described.^33^ In essence, trained psychologists conducted interviews with these BD patients using the Diagnostic Interview for Genetic Studies (DIGS). The inclusion criteria for BD patients required them to participate in the study only after a 4‐week period of stable medication use and achieving euthymic status, defined as a Young Mania Rating Scale (YMRS) score of <6 and a Montgomery‐Åsberg Depression Rating Scale (MADRS) score of <13.

A total of 18 offspring (BDoff), each having one parent affected by BD, were recruited. The parents of the proband were outpatient individuals receiving care for BD at the Mood Clinic at Geneva University Hospital's Psychiatry Department. To minimize potential family‐related biases, all study participants were unrelated.

A total of 53 healthy control participants (HC) were recruited using web announcements or local databases. All participants, under the ethical approval CER 13‐081 granted by Geneva University, provided written informed consent. Prior to the MRI session, clinical questionnaires, detailed in Data S1, were completed by all subjects.

### 2.2 MRI data acquisition and analysis

Details of whole‐brain, resting‐state fMRI data acquisition, preprocessing and seed‐based data analysis for all participants are provided in Data S1. For the seed selection, we utilized a comprehensive parcellation of the entire bilateral hippocampus, supported by anatomical (Harvard Oxford atlas) and functional connectivity (task‐related and resting‐state) data, validated through meta‐analytic connectivity mapping from two distinct databases (neurosynth and brainMap).^12^, ^13^

We then implemented the μCAPs analysis, which allows to pinpoint the contribution of different sub‐sections of the seed region to specific pattern of synchronous brain activity, that is, co‐activation patterns (CAPs). The μCAPs analysis is an extension of the well‐established CAPs analysis, which has been extensively described in previous studies^20^, ^30^ and is detailed in Data S1. Unlike CAPs analysis where the seed is considered homogeneous, μCAPs analysis applies a data‐driven approach to identify differentiation within the seed for each candidate brain pattern. This iterative approach allows for the identification of K μCAPs, each associated with a weight map for the seed. The code for μCAPs analysis is available at: https://github.com/MIPLabCH/mCAP and it is described in detail elsewhere,^32^ and in Data S1. This allows a finer hippocampal characterization, pinpointing which of its subdivisions contributes to each dynamic brain pattern (or μCAPs).

In total, five μCAPs strongly co‐activating with five hippocampal subdivisions were identified (Table S1), based on a data‐driven “test–retest” procedure, detailed in Data S1. This procedure indicated K = 5 as the optimal number of distinct brain clusters in terms of replicability for this dataset.

Frames assigned to each μCAP were traced back to the individual's time‐course and occurrences of each μCAPs within participants were calculated and extracted for further analysis. Occurrences, in this context, represent the cumulative time‐points (frames) associated with specific μCAPs across the entire time‐course. In simpler terms, occurrences signify how frequently a subject's brain activity matched a particular spatial configuration indicated by the μCAPs, while the corresponding seed hippocampal subregion was also active. Details on motion correction and sub‐analyses to exclude motion as a potential confounding factor are provided in Data S1 (Sections 1.5 and 2.4, respectively).

### 2.3 Statistical analyses

In our analysis, we utilized linear mixed models (LMM),^34^ which were implemented in R (https://www.R‐project.org/). These models were employed to conduct a comparative assessment of the normalized occurrences of μCAPs across different groups, namely BD, BDoff, and HC. We controlled for various factors, including sex, age, medication usage, and multiple clinical scores (further details in Data S1). The final base LMM, modeling “sex” and “age” as fixed effects and including a random effect across subjects, was:

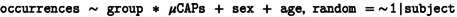




The visual examination of residual plots did not detect any deviations from normality or homoscedasticity. To obtain the *p*‐values, likelihood ratio tests (ANOVA) were conducted, comparing the full model with the effect under investigation to a model that lacks this effect. To explore the relationships and interactions between μCAPs and BD symptoms, we ran Spearman's rank partial correlations^35^ between μCAPs occurrences and the clinical scores, and we compared correlations between BD, BDoff, and HC with the R‐based *Cocor* package.^36^ Results were adjusted for age, sex, and multiple comparisons using the false discovery rate (FDR) or the Tukey HSD (honestly significant difference) test, as appropriate.

## 3 RESULTS

### 3.1 Clinical scores and demographics

As described in the Methods, the study comprised 97 participants, including 26 euthymic bipolar disorder (BD) patients (11 patients suffering from BD type 1, 14 from BD type 2, and one from BD not otherwise specified). On average, BD patients had experienced 8 lifetime mood episodes (standard deviation, SD: 7). Groups were matched for gender and education. Table 1 provides an overview of the other main clinical and demographic characteristics of the participants.

**TABLE 1:** Participants' demographic and clinical characteristics.

	BD patients (*N* = 26)	BD offspring (*N* = 18)	Controls (*N* = 53)
Demographics			
Age: mean (SD)	32.7 (12.2)	19.7 (3.1)	25.3 (9.5)
Females/males	13/13	9/9	23/30
Education, mean (SD)	14 (3.4)	13 (2.3)	14.2 (3.1)
Clinical			
BD type 1/2	12/14	NA	NA
ALS: mean (SD)	1.1 (0.7)	0.6 (0.5)	0.4 (0.3)
MADRS: mean (SD)	3.8 (3.4)	1.9 (2.4)	1.1 (1.7)
YMRS: mean (SD)	1 (1.5)	0 (0)	0 (0)
CERQ (non‐adaptive): mean (SD)	52.5 (28.6)	43.4 (22.3)	39.2 (21.2)
RRS: mean (SD)	24.1 (5.6)	20.4 (5.5)	20.1 (5.5)
Disease severity			
Number of episodes: mean (SD)	8 (7)	NA	NA
Disease duration, mean (SD)	14 (10.7)	NA	NA
Hospitalizations: mean (SD)	4.1 (3.9)	0	0

*Note*: The table recapitulates the main demographic and clinical characteristics of BD patients and healthy controls.

Abbreviations: ALS, Affective lability scale; BD, bipolar disorder; BDoff, bipolar disorder patients' offspring; CERQ, non‐adaptive subscore of the emotion regulation questionnaire; HC, healthy controls; MADRS, Montgomery–Åsberg Depression Rating Scale; NA, not applicable; RRS, Ruminative Response Scale; SD, standard deviation.

Notably, BD patients exhibited significantly higher scores on various clinical scales even during euthymic state. Specifically, individuals with BD scored higher than those in the HC group on several measures, including the non‐adaptive section of the emotion regulation questionnaire (CERQ), the Montgomery–Åsberg Depression Rating Scale (MADRS), the affective lability scale (ALS), and the short version of the Ruminative Response Scale (RRS) (*p* < 0.05 for all tests). These findings indicate that even when not experiencing active mood episodes, BD patients still demonstrated greater depressive symptoms, affective lability, rumination tendencies, and maladaptive emotion regulation strategies compared to the control group. On the other hand, no differences in other clinical scores between BDoff and the other two groups were identified (*p* > 0.05 for all comparisons). While the majority of BDoff did not have any psychiatry diagnosis, 33% of BDoff had one or more of these psychiatric diagnoses: history of depression (*n* = 2), panic disorder (*n* = 1), PTSD (*n* = 1), eating disorder (*n* = 1), ADHD (*n* = 3). This confirms that our BDoff sample was overall representative, since it is well‐documented that BDoff present a high lifetime prevalence of psychiatric disorders.^37^

Further details on sub‐analyses to exclude a confounding effect of laterality, sex, cognitive and clinical scores, medication, movement, age, and probability of transition between μCAPs are provided in Data S1.

### 3.2 Micro‐coactivation patterns identification

In our study, we identified five μCAPs using the algorithm described in the Methods, each connected with a specific hippocampal subfield (Table S1). These five μCAPs corresponded to well‐characterized resting‐state networks, based on existing literature,^38^, ^39^ as follows: a frontoparietal μCAP (FPN, also known as the central executive network, CEN, or the executive control network) involved brain regions such as the superior parietal lobule, the temporal complex, and frontal eye fields. A somatomotor‐μCAPs (SMN) encompassed pericentral and visual areas. A limbic network‐μCAP (LN) involved the orbitofrontal cortex, amygdala, temporal insula, and ventral tegmental area. A DMN‐μCAP (also known as medial frontoparietal network) includes the prefrontal cortex, the posterior extent of the inferior parietal lobule, the precuneus, and the posterior cingulate cortex.^38^, ^39^

Finally, a saliency‐network‐μCAP (SN, also known as the salience network, midcingulo‐insular network, or cingulo‐opercular network) involved the anterior midcingulate cortex and bilateral insula.^38^, ^39^

Data‐driven analysis of fMRI data showed that, across all 97 participants, each of these large‐scale brain networks was associated univocally with a specific data‐driven hippocampal subdivisions, as detailed in Table S1. The FPN, responsible for cognitive and executive control and attentional processes, was linked to the lateral body of the hippocampus. The SMN, responsible for governing sensorimotor functions, was associated with the anterior body of the hippocampus. The LN, known for its involvement in emotion processing and regulation, was associated with the hippocampal head. The DMN, responsible for introspective and self‐referential cognitive activities, is associated with the medial body of the hippocampus. Lastly, the SN, critical for detecting and prioritizing significant stimuli, demonstrated a correlation with the hippocampal lateral tail.

### 3.3 Differences in temporal dynamics of hippocampal subfields dFC

**FIGURE 1:**
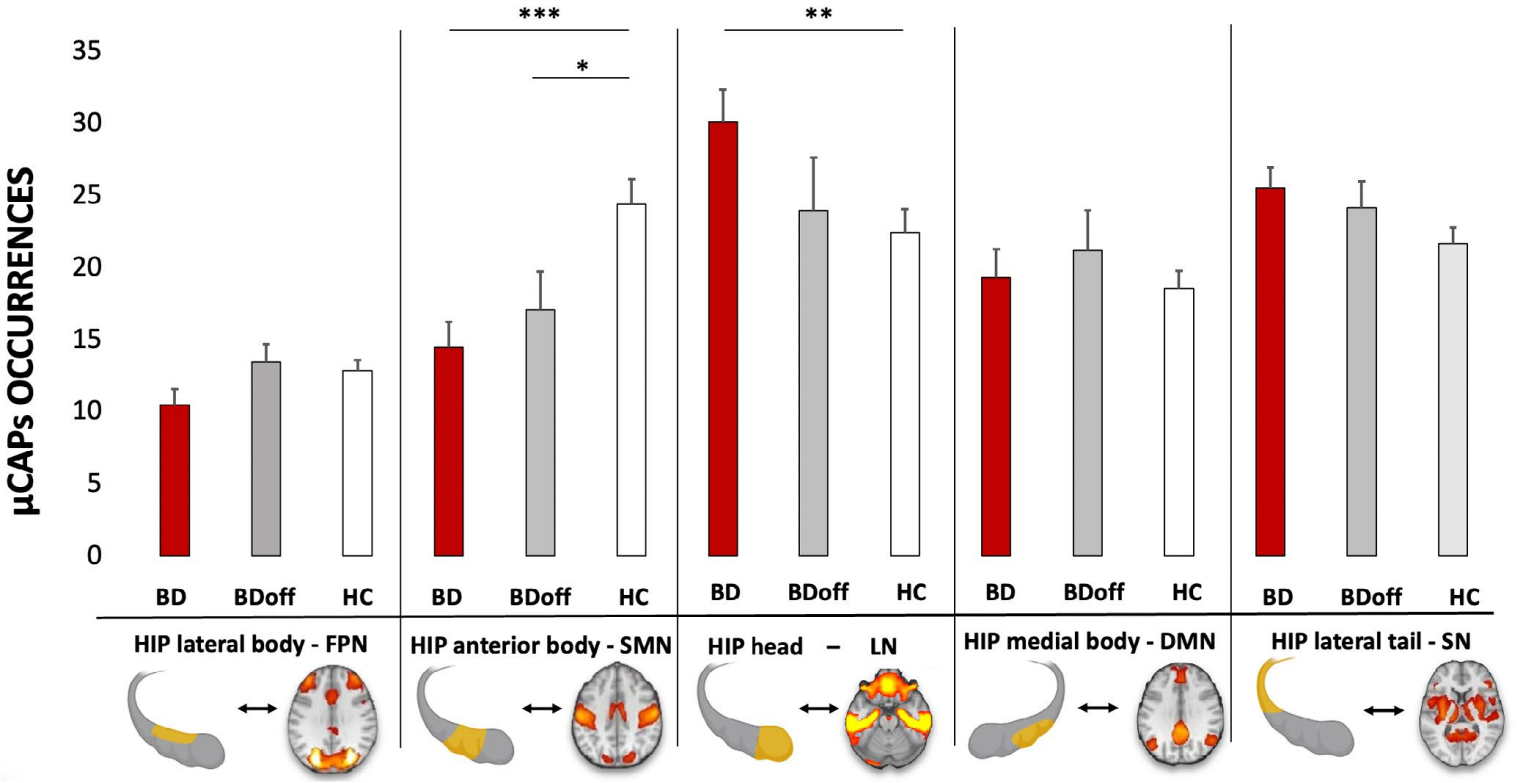
Differences in temporal dynamics of hippocampal dFC. We identified five micro‐CAPs (μCAPs) using the bilateral hippocampus (HIP) as seed: A frontoparietal μCAP (FPN) associated with the hippocampal lateral body, a somatomotor‐visual μCAP (SMN) associated with the hippocampal anterior body, a limbic network μCAP (LN) associated with the hippocampal head, a default‐mode network μCAP (DMN) associated with the hippocampal medial body, and a saliency‐network μCAP (SN) associated with the hippocampal lateral tail. The normalized occurrences of the SMN‐μCAP were significantly lower in individuals with BD (bipolar disorder) patients and offspring of BD patients (BDoff) than in HC (healthy controls). The occurrences of a limbic‐μCAP were significantly more frequent in BD patients than in HC. Three stars indicate a significance level of *p* < 0.0005; two stars indicate a significance level of *p* < 0.005; one star of *p* < 0.05, all adjusted for FDR (false discovery rate), sex, and age.

The occurrences of the SMN‐μCAP were significantly less frequent both in BD patients (β: −9.97, Standard Error, SE: 2.34, Degree of Freedom, DF: 92, *t*: −4.25, *p*‐value_FDR_: 0.00015) and in BDoff (β: −7.26, SE: 2.65, DF: 92, *t*: −2.73, *p*‐value_FDR_: 0.020), than in HC (Figure 1).

The occurrences of the LN‐μCAP were significantly more frequent in BD patients than in HC (β: 7.66, SE: 2.4, DF: 92, *t*: −3.18, *p*‐value_FDR_: 0.005). Once again, BDoff presented an intermediate pattern of dFC (mean: 30.1, SE: 2.2), in between BD (mean: 24, SE: 3.6) and HC (mean: 22.4, SE: 1.6), although not significantly different (Figure 1).

Finally, we report a non‐significant trend in the occurrences of the SN‐μCAP, which was more frequent in BD (mean: 25.6, SE: 1.3) than in BDoff (mean: 24.1, SE: 1.8), and more frequent in BDoff than in HC (mean: 21.6, SE: 1.1), with an interesting gradient between the three groups (Figure 1).

### 3.4 Interactions between hippocampal subfields dFC networks and clinical symptoms

**FIGURE 2:**
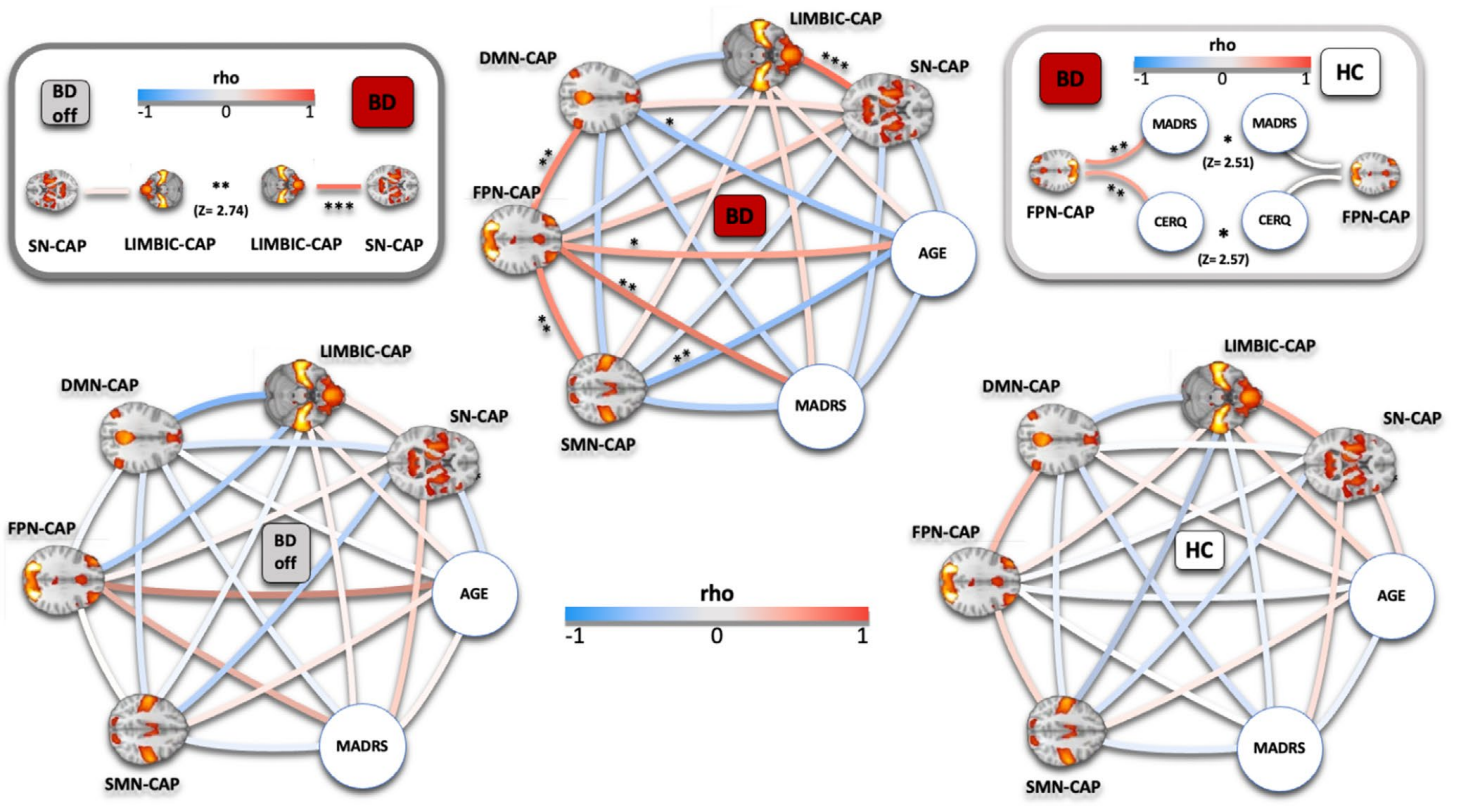
Interactions between hippocampal dFC networks and symptoms. The interactions between the somatomotor‐visual CAP (SMN‐μCAP), frontoparietal CAP (FPN‐μCAP), default mode network CAP (DMN‐μCAP), saliency‐network CAP (SN‐μCAP), and depression scores (MADRS) are disrupted in individuals with BD (bipolar disorder, top center) compared to HC (healthy controls, bottom right) and BDoff (offspring of BD patients, bottom left), adjusting for age. SN‐μCAP and LN‐μCAP were significantly more correlated in BD patients compared to BDoff (top left). FPN‐μCAP was significantly correlated with depressive symptoms (MADRS) and non‐adaptive emotion regulation score (CERQ) in BD patients. These correlations were significantly stronger in BD patients than in HC (top right). The color of the connections indicates the strength of the positive (red) or negative (blue) correlations, according to the Spearman's rho. Three stars indicate a significance level of *p* < 0.0005; two stars indicate a significance level of *p* < 0.005; one star of *p* < 0.05, all adjusted for FDR (false discovery rate).

We present here the partial correlation analysis among μCAPs (seeded at the bilateral hippocampus), including MADRS and age (Figure 2). There were no significant correlations in the BDoff and in the HC groups. On the contrary, there were multiple significant correlations in the BD group, and we describe here the most relevant ones.

In BD patients, both non‐adaptive subscores of the CERQ (not shown) and MADRS were positively correlated with FPN‐μCAP occurrences (*p*‐value_FDR_ < 0.005). Concerning interactions between networks in BD patients, FPN‐μCAP was positively correlated both with DMN‐μCAP and SMN‐μCAP occurrences (*p*‐value_FDR_ <0.005) for both correlations. LN‐μCAP was positively correlated with SN‐μCAPs occurrences (*p*‐value_FDR_ < 0.0005).

We then investigated whether any correlation was significantly different between groups. The positive correlation between LN‐μCAP and SN‐μCAP was significantly higher in BD compared to BDoff (*p*‐value_FDR_ < 0.005, Figure 2). The positive correlations between FPN‐μCAP and the clinical scores (MADRS and non‐adaptive CERQ) were significantly higher in BD compared to HC (*p*‐value_FDR_ < 0.05 for both comparisons, Figure 2).

Other secondary and non‐significant findings on μCAP temporal dynamics and their interactions are detailed in Data S1.

## 4 DISCUSSION

### 4.1 μCAPs activity‐based hippocampal weight maps corroborate existing functional and structural subdivisions of the hippocampus

As discussed in the Introduction, research on behavioral, structural, and functional profiling has highlighted the existence of an “emotion‐cognition gradient” along the anterior–posterior axis of the hippocampus, with the hippocampal head being more prominently involved in emotion regulation processing.^12^, ^13^ Our study's findings align with this concept. Indeed, our agnostic, data‐driven differentiation within the hippocampus as a seed revealed that the head was associated with activity in limbic brain areas that are known to be involved in emotion processing (specifically, the LN). Likewise, previous research has shown that the anterior body of the hippocampus is involved in processing external somatomotor information.^5^ This observation is consistent with our findings, as the emergence of the hippocampal anterior body within the seed was associated with the SMN. It is not surprising that the SMN should be connected with part of the anterior hippocampus (i.e., the subdivision most involved in affective processing) considering that the SMN has been implicated in emotion regulation, as discussed below.^21^, ^22^, ^23^, ^24^, ^25^, ^26^, ^28^, ^40^

Additional findings on the associations between data‐driven within‐seed weight maps and large‐scale brain networks are discussed in Data S1 and in Table S1.

### 4.2 Differences in temporal dynamics of hippocampal uCAPs

#### 4.2.1 Reduced dynamic functional connectivity of the hippocampal anterior body with the SMN: a vulnerability marker in BD offspring?

The results of our study indicate that both BD patients and BDoff exhibited reduced dFC between the hippocampal anterior body and the SMN compared to HC. These results agree with previous evidence showing that activity in the anterior hippocampal body is correlated with SMN.^5^ Additionally, our results converge with and expand upon our previous research using a less recent dFC analysis technique (i.e., conventional CAP analysis^30^), which also demonstrated reduced hippocampal dFC with the SMN in a subset of BD patients.^31^ Of particular interest is our novel finding that BDoff displayed an intermediary pattern of dFC, positioning themselves between BD patients and HC. This intriguing observation suggests that altered hippocampal dFC with the SMN could serve as a meaningful indicator of vulnerability to BD.

Indeed, accumulating evidence from various modalities of FC analysis points to SMN disruption in BD patients.^20^, ^26^, ^27^, ^31^, ^41^ Multiple interpretations of these SMN alterations exist.

As mentioned in the Introduction, recent research has increasingly implicated the SMN in emotional processing.^28^ The SMN is necessary for emotion recognition (the first step of emotion regulation), and fMRI evidence points to the role of SMN in the generation of emotional responses as well as in interoceptive attention.^28^ Additionally, the SMN is involved in multiple emotion regulation strategies such as situational selection and attentional deployment, for instance by modulating saliency assignment and tactile attention.^28^ Thus, considering the role of SMN impairment in emotion dysregulation and evidence suggesting its role in emotional processing specifically in BD,^21^, ^22^, ^23^, ^24^, ^25^, ^26^, ^40^ we propose that the reduction in SMN dFC with the anterior hippocampal body may be related to impaired sensory processing and altered assignment of saliency to sensorimotor cues in BD. This may underlie emotion dysregulation in BD, and vulnerability in BDoff to emotion dysregulation disorders, such as BD.

The non‐significant trend in the dFC between the hippocampal tail and the SN, which progressively decreased from BD to BDoff to HC, further supports the idea that anomalies in salience processing could complement the SMN alterations, contributing to vulnerability to emotion dysregulation.^28^ This agrees with our previous significant finding of increased dFC between the left hippocampus and the SN in BD patients.^31^ Moreover, aberrant saliency assignment is considered a marker of high risk for psychosis.^42^

An additional interpretation of SMN hypoconnectivity in BD takes into account the fact that reduced psychomotor activity and somatomotor dulling characterize depressive states,^43^ whereas (hypo)manic states, involving motor agitation and hyperesthesia, associate with SMN hyperconnectivity, particularly when psychomotor agitation is predominant.^44^ Considering that depressive symptoms often persist in euthymic BD patients and that these symptoms may be an expression of emotion dysregulation,^45^ especially in individuals at risk such as BDoff, it is plausible that BD patients and BDoff could still present reduced psychomotor activity or increased vulnerability to depressive symptoms, leading to lower dFC between the hippocampal anterior body and the SMN.

#### 4.2.2 Increased dynamic functional connectivity of the hippocampal head with the limbic network in BD patients

dFC between the hippocampal head and the LN was higher in BD patients than in HC. BDoff, once again, displayed an intermediate pattern of dFC between BD and HC, although the difference was not statistically significant. This suggests an hyperactivation of emotion‐processing circuits involving the anterior hippocampus, orbitofrontal cortex (OFC), and amygdala in BD patients, without corresponding activation of the lateral prefrontal modulatory cortical regions (such as DLPFC and VLPFC). This pattern is widely recognized as a marker of emotion dysregulation.^6^, ^46^, ^47^

More specifically, heightened amygdala‐OFC FC is a purported trait of vulnerability to depression.^48^ This aligns with the finding of reduced SMN activity in both BD patients and BDoff, potentially signifying an increased vulnerability to residual depressive symptoms, as discussed in the previous section.

Therefore, our study highlights, for the first time, the involvement of the anterior hippocampus specifically in the disruption of sensorimotor and limbic processing, not only in BD patients but also in BDoff. Considering the well‐established evidence of hippocampal abnormalities in BD ^7^, ^8^ and its multiple roles in emotion, cognition, and sensory processing, the hippocampus appears to play a crucial role in BD symptomatology. Our findings suggest that hippocampal abnormalities in BD patients might contribute to disrupted activity in the SMN and LN, in agreement with existing research,^20^, ^26^, ^41^ and to the vulnerability to emotion dysregulation disorders in BDoff.

### 4.3 The interactions between hippocampal dFC networks are modulated by BD symptoms and distinguish BD patients from offspring of BD patients

Concerning clinical scores, BD had significantly higher depressive and emotion dysregulation symptoms than HC, consistent with the well‐known high prevalence of residual symptoms in euthymic BD patients.^49^

The data‐drive differentiation within the hippocampus through the mCAPs technique allowed to identify large‐scale networks associated with specific hippocampal subregions. Therefore, to explore the relationship between these hippocampal dFC networks and depressive and emotion dysregulation symptoms (Figure 2), we conducted a between‐network correlation analysis, revealing widespread large‐scale network dysfunctions in BD patients. Indeed, only in BD patients was the FPN significantly correlated with both the DMN and the SMN. Furthermore, the positive correlations between FPN and both depressive and emotion dysregulation symptoms were significantly higher in BD patients than in HC (Figure 2). This is particularly interesting when considering that the FPN was connected with the hippocampal Cornus Ammonis (CA1) in our analysis, which has been specifically implicated in depression in previous research.^50^ Additionally, the anti‐correlation between the DMN and FPN is crucial for cognitive control and adaptive mood and emotion regulation,^21^ as it allows for flexible redirection of attention away from internal processes and toward external cues.^51^ Thus, we hypothesize that the significant correlation between the DMN and FPN activity in BD represents a reduced ability to switch neural resources from one network to another, contributing to emotion dysregulation, depressive symptoms and disproportionate involvement of internally oriented attentional processes, in agreement with existing literature.^33^, ^52^ These findings align with existing literature that has implicated the FPN in internally focused attention, cognitive control, and executive function,^38^ which are closely related to sensorimotor processing. In fact, impaired SMN FC correlates with clinical symptoms and disrupted executive function in BD patients,^22^ which agrees with our finding of a significant positive correlation between SMN and FPN in BD patients. This correlation indicates aberrant attentional and executive processing of sensory information by the FPN, whose activity indeed correlates with emotion dysregulation symptoms in BD patients only. This interpretation is further supported by the strong positive correlation between LN and SN, which differentiates BD patients from BDoff (Figure 2). This suggests that, in BD patients, the dysregulation of emotion‐processing networks is tightly linked with dysregulation of saliency‐processing ones, and this may mark the transition from a state of vulnerability to BD (in BDoff) to clinical BD (in BD patients), while SMN alterations may be present in BDoff before the development of full‐blown BD.

In sum, the increased correlation observed between large‐scale functional brain networks in BD patients may underlie a lower ability to switch neural resources from one network to another (FPN), resulting in increased self‐referential thoughts (DMN), and disrupted somatomotor (SMN) and emotion (SN and LN) processing. Importantly, the dysregulation of emotion‐ and saliency‐processing networks may serve as vulnerability markers for BD in high‐risk individuals. Furthermore, our results indicate that some BD patients, who would be classified as “euthymic” based on clinical scores, still experience lingering depressive and emotion dysregulation symptoms impacting their cerebral activity.

### 4.4 Strengths and limitations

Strengths of our study include the novel dFC technique (μCAPs) used to explore hippocampal subfields dFC in BD patients and BDoff, yielding significant advantages compared to existing FC studies in BD. Additionally, the inclusion of both BD patients and BDoff sheds light on potential patterns of vulnerability to BD in high‐risk individuals. Sub‐analyses did not identify significant effects of potential confounding variables, providing further robustness to our findings. However, our study also has limitations, including the sample size that hampered conclusive sub‐analyses, and the inability to formally advance hypotheses on markers of BD state or subgroup differences. Despite the sub‐analyses of the main confounding variables, potential confounding factors such as medication status, hospitalizations, comorbidities, and substance use may remain sources of heterogeneity. Additionally, limitations intrinsic to our μCAPs analysis include the a‐priori definition of the initial region of interest (i.e., the hippocampus), and of the threshold for selecting frames, which is however in agreement with existing studies on the CAPs method.^29^, ^30^, ^53^, ^54^ While dFC markers are extremely promising as therapeutical markers thanks to their non‐invasive nature and to the correlations with BD symptoms, fMRI remains an expensive and relatively time‐consuming assessment. Future research should address these limitations and potentially explore different cerebral seed regions (such as the left or right hippocampus only, instead of the bilateral hippocampus) to uncover other significant differences in BDoff that appear as non‐significant trends in our results.

### 4.5 Conclusion

The present study revealed alterations in large‐scale functional brain networks in BD patients, associated with disruptions in cognitive control switching (i.e., in the FPN) correlating with emotion dysregulation and depressive symptoms, as well as with increased involvement of internally oriented attentional processes (DMN) and disrupted somatomotor (SMN). The application of μCAPs dFC analysis allows to differentiate within the seed region contributions of hippocampal subdivisions to SMN and LN in BD. Notably, BDoff demonstrated an intermediate phenotype between BD and HC, indicating, for the first time, that dFC of hippocampal subdivisions, particularly with the SMN and emotion processing networks (i.e., SN and LN), may serve as a marker of vulnerability to BD in high‐risk individuals.

As a perspective, such neuroimaging markers of BD physiopathology and of vulnerability to BD may guide early interventions, to investigate whether modulations of these markers in individuals at high risk for BD may reduce the risk of progression to full‐blown BD, or may alleviate the burden of prodromal symptoms. Different targets of such interventions have been proposed. For instance, since a pro‐inflammatory state may be associated with BD,^17^, ^55^ and inflammation has been suggested to specifically impact emotion regulation neural circuits in BD,^27^, ^56^, ^57^ potential early interventions may speculatively target this pro‐inflammatory state in BD patients and offspring. Another avenue worth exploring, as a perspective, maybe the use of dFC of hippocampal subdivisions as therapeutic markers for mindfulness‐based cognitive therapy. Indeed, a study involving 22 BD patients demonstrated that mindfulness‐based cognitive therapy restored the normal anti‐correlation between the DMN and control networks, which is crucial for the ability to flexibly redirect attention from internal processes to the external environment.^51^ Finally, our findings on hippocampal dFC correlates of residual depressive and emotion dysregulation symptoms open avenues for further investigation into how these neuroimaging markers may offer clinicians a valuable tool to objectively quantify the impact of residual symptoms on euthymic BD patients' cerebral activity and functioning, potentially enhancing patient stratification in research settings or therapeutic interventions, as well as prognostic assessments.

In conclusion, our results underscore the need for further research to characterize the balance between large‐scale brain networks as a potential marker for BD. The insights gained emphasize the complexity of BD and advocate for a comprehensive assessment of its neural underpinnings. Overall, this study contributes to advancing our understanding of BD and provides a foundation for future research aimed at improving the prognosis for individuals living with this complex disorder, and at the identification of neuroimaging markers associated with vulnerability to BD in high‐risk subjects, potentially enhancing early interventions.

## AUTHOR CONTRIBUTIONS

LFS: Writing—Original Draft, Visualization; LFS, CP, FD, DVDV: Conceptualization, Writing—Review & Editing; LFS, FD: Methodology, Investigation, Formal Analysis, Data Curation; LFS, CP: Funding Acquisition; CP, DVDV: Resources, Supervision.

## FUNDING INFORMATION

This work was supported by the Swiss National Center of Competence in Research (NCCR); “Synapsy: the Synaptic Basis of Mental Diseases” financed by the Swiss National Science Foundation [Grant Number 51NF40‐158776], as well as a grant of the Swiss National Science Foundation [Grant Number 32003B_156914], and two scholarships, one by NCCR‐Synapsy and one by the Frutiger‐Bickel foundation.

## CONFLICT OF INTEREST STATEMENT

None of the authors has any financial disclosure or conflict of interest.

## Supporting information


Data S1.

## Data Availability

The data that support the findings of this study are available from the corresponding author upon reasonable request.
