# Supplementary material for: Hippocampal temporal dynamics and spatial heterogeneity unveil vulnerability markers in the offspring of bipolar patients
Source: Bipolar Disord. 2024 Aug 12;27(1):17–27. doi: 10.1111/bdi.13487 (PMC11848017; doi:10.1111/bdi.13487)
Supplement: Supplementary file 1 — Data S1. [file BDI-27-17-s001.docx]

**SUPPORTING INFORMATION**

**Hippocampal temporal dynamics and spatial heterogeneity**

**unveil vulnerability markers in offspring of bipolar patients**

**Authors**

Luigi F Saccaro, MD^a,b,d^; Farnaz Delavari, MD^c,d^; Dimitri Van De Ville, PhD^d,e^; Camille Piguet, MD, PhD^a,f^

**Affiliations**

^a^ Psychiatry Department, Faculty of Medicine, University of Geneva, Switzerland

^b^ Psychiatry Department, Geneva University Hospital, Switzerland

^c^ Developmental Imaging and Psychopathology Laboratory, University of Geneva School of Medicine, Geneva, Switzerland

^d^ Medical Image Processing Laboratory, Institute of Bioengineering, École Polytechnique Fédérale de Lausanne, Lausanne, Switzerland

^e^ Institute of Bioengineering, School of Engineering, Ecole Polytechnique Fédérale de Lausanne (EPFL), Geneva, Switzerland

^f^ Child and Adolescence Psychiatry Division, Geneva University Hospital, Switzerland

**Running title:** Hippocampal markers of bipolar disorder risk

***Corresponding author:** Luigi Francesco Saccaro, MD, Psychiatry Department, Faculty of Medicine, University of Geneva, Switzerland, Psychiatry Division, Geneva University Hospital, Rue Gabrielle-Perret-Gentil 4, 1205 Geneva, Switzerland; email: [LuigiFrancesco.Saccaro@unige.ch](mailto:LuigiFrancesco.Saccaro@unige.ch)

1. SUPPLEMENTARY METHODS
   1. Further description of clinical evaluation of the participants

Before the scanning session, all subjects completed the Montgomery-Asberg Depression Rating Scale, the Young Mania Rating Scale (YMRS), the affective lability scale (ALS)(1), the non-adaptive section of the emotion regulation questionnaire (CERQ)(2), and a short version (10 items) of the Ruminative Response Scale (RRS)(3). Other form of psychosis such as schizo-affective disorders were an exclusion criterion. The presence of an intellectual disability was excluded thanks to the Diagnostic Interview for Genetic Studies (DIGS, as described in the Methods), which includes a scoring for intellectual functioning, comorbidities, and attention. Additionally, we collected measures of working memory (such as mental calculation, forward and reverse digit span). All these assessments did not highlight cognitive impairment nor intellectual disability in our participants.

- 1. Further description of MRI data preprocessing

Structural and functional images were preprocessed in MATLAB 2018a (MathWorks, Inc., Natick, MA, USA) with a previously published pipeline(4) using SPM12 (Wellcome Trust Centre for Neuroimaging, London, UK; <http://www.fil.ion.ucl.ac.uk/spm>). Briefly, functional images were realigned, followed by spatial smoothing using a 3 mm full width at half maximum (FWHM) isotropic Gaussian kernel. The structural scans were co-registered to the mean functional image. The structural images were segmented using SPM12's segmentation algorithm(5), and Diffeomorphic Anatomical Registration using Exponentiated Lie algebra (DARTEL)(6) was used to create a study-specific template. The first five functional scans were excluded to allow for magnetization equilibration. To remove confounding effects, linear detrending was applied to the time series, and the motion parameters (translation and rotation) along with global, mean white matter, and mean cerebrospinal fluid signals were regressed out using the warped DPARSF tissue masks(7). The time series were then bandpass filtered to 0.01-0.10 Hz. After preprocessing, all functional images were warped to the DARTEL space and subsequently to MNI space for population-based analysis. Additionally, the µCAPs algorithm included extended motion correction by identifying frames with high framewise displacement (FD > 0.5 mm(8)) and excluding them from the analysis (see Sections 1.5. and 2.4. for further details on motion).

- 1. ***Basic principles of CAPs analysis***

CAPs analysis disentangles different functional brain networks interacting with the seed region through a spatio-temporal frame-wise fMRI deconvolution. This approach conceptually agrees with the model of discrete transitions between brain meta-states(9) and its physiological soundness is supported by multiple works, as previously described(10). Compared with stationary connectivity analyses, which neglect time-varying data, CAPs analysis captures neural activity fluctuations to cluster also spatio-temporally overlapping patterns of brain activation, which are thus not treated as mutually exclusive. Further, compared with other methods of dFC analysis, CAPs focus on single fMRI volumes at individual time points, instead of fMRI time courses, and are thus highly sensitive in identifying and analyzing brief, recurring, patterns of co-activation and their temporal variability(10,11).

CAPs analysis has been extensively described in previous studies(11,12) . Briefly, CAPs analysis extracts and Z-scores the average seed BOLD signal across the fMRI time-series, selecting the time points with “higher than threshold” activity. Subsequently, a K-means clustering algorithm is applied to agnostically classify the retained whole-brain volumes from all subjects into distinct clusters representing the CAPs, for which within-cluster differences (defined as spatial similarity) were smaller than across-cluster ones (more details about clustering are provided in(13). These CAPs are then converted into spatial Z-maps, to quantify the significance of their deviation from zero.

- 1. ***Details of μCAPs analysis***

1. *Initialization and seed focalization*

As described in detail elsewhere (14), μCAPs analyze BOLD fMRI data employing a matrix that represents the time-dependent fMRI volumes for all subjects, in voxels. A subset of the voxels is defined as the seed, or the region of interest, in this case the bilateral hippocampus. The goal is to identify a number (“*K”)* of micro co-activation patterns (µCAPs) associated with weight maps for the seed ROI. At the first step, the arithmetic mean of BOLD signal within the voxels of the initial seed is calculated and used as the first time-course for frame-selection. At following iterative steps, the time course for each seed weight map is computed by averaging the fMRI volumes weighted by the corresponding seed weights. Therefore, allowing smaller seed regions to have an equally great role in frame-selection in the next steps.

*b) Criteria for selection of frames*

In this iterative process, a threshold for the Z-scored time course for each seed weight map is defined, and if activity exceeds this threshold, the corresponding fMRI volumes are tagged as "selected." The initial threshold is set to 0.5 for maximum sensitivity, and in subsequent iterations, it is set to 1.0, a typical value used in conventional CAP analyses (15,16).

*c) Clustering*

All fMRI volumes selected based on the seed activation are clustered by a K-means algorithm on a 1-correlation distance metric. The centroid of each K-cluster represents a micro co-activation pattern (µCAP). The K-means is done for candidate number of clusters ranging from (K = 2 : 10). The optimal number of clusters (K) is determined through a test-retest algorithm including a random split of subjects into train and test subgroups described in detail elsewhere (14). Briefly, the train frames go through K-means clustering to obtain K centroids. The distance of the test frames to the K training centroids is calculated. The cluster with the highest sum of distances of test frames is chosen as the worst-fit cluster and is monitored across the range of candidate K values (2 : 10). While the sum of distances decreases as K increases, a local drop in the sum of distances for the worst-fit cluster indicates an optimal fit for a specific candidate K, which is thus selected.

*d) Seed weight maps update*

To update the seed weight maps for the next iteration, each µCAP is restricted to the seed region and its values are assigned to the corresponding seed weights. We adopt a "winner-takes-all" approach by keeping only the largest µCAP value for each voxel and setting values for other µCAPs to zero. The algorithm then iterates, going to frame-selection again, until convergence is achieved.

*e) Convergence*

Convergence was defined as the point for which the distance between the seed patterns of matching μCAPs of the previous and the new step were less than 0.05. The distance is computed using the Hungarian algorithm(17) and averaged cosine distance across iterations. Convergence is considered achieved when the distance falls below a threshold of 0.005. The first iteration (in which the seed pattern is uniform), is not included in the convergence assessment.

- 1. ***Motion correction***

In addressing potential motion artifacts, our methodology involved a two-step process. Firstly, subjects exhibiting excessive translational or rotational motion (pre-defined as exceeding 3 mm), were excluded from the analysis. One subject was removed at this stage, leading to the final number of 97 subjects. Additionally, to make sure the patterns of μCAPs are not derived from frames that are disrupted by movement, we employed a scrubbing method following established protocols (8). Frames with a frame-wise displacement (FD) above 0.5, calculated using the Power formula (8), were systematically removed to mitigate the impact of motion-related artifacts.

- 1. ***Figures and graphs***

The brain networks were visualized with Mango image processing software (Lancaster, Martinez; www.ric.uthscsa.edu/mango). Other graphs and figures were generated using Matlab©, Excel, PowerPoint, Biorender, and R©.

1. **SUPPLEMENTARY RESULTS**
   1. ***Sub-analyses on laterality***

Besides our base linear mixed effects model (LMM), an additional LMM was run to determine whether laterality influenced the results. It used the base model with the addition of a binary predictor for laterality (left-handed, n=13, or right-handed, n=84). The addition of laterality, besides age and sex, to the base model did not change the significant results. Furthermore, the number of left-handed subjects was not significantly different between groups (p>0.9 for all comparisons).

- 1. ***Sub-analyses on sex ratios and working memory***

There was no significant difference between the three groups for sex ratios (p>0.8 for all comparisons), nor for working memory indices, such as direct digit span memory, reverse digit span memory, ascending digit span memory, or mental calculations (p>0.5 for all comparisons between groups).

- 1. ***Sub-analyses on medication***

While the vast majority of BD patients had at least one psychotropic medication, only two of the BDoff were under treatment (antidepressants). All the significant differences in uCAPs occurrences between groups reported in the main text remained significant after repeating the analyses without these two BDoff (p<0.05 for all comparisons). HC were not taking psychotropic treatments, the only treatments reported among them were contraceptive pill (n=1) and probiotics (n=1).

Considering this obvious difference among groups, and to further explore the effect of medications, total psychotropic treatment load was calculated for each participant (zero, one, or more psychotropic medications). This variable was then added to the base model to adjust for medication load in the analysis, including sex and age as covariates, by the following R-based LMM formula syntax:

occurrences ~ group * μCAPs + sex + age + medication_load, random=~1|subject

where the independent variable “group” refers to the participants’ groups (BD, BDoff, HC) and “μCAPs” to each μCAP. Additionally, “sex” and “age” and the medication load (“medication_load”) were modeled as fixed effects, while “subjects” included a random effect across subjects. Visual inspection of residual plots did not reveal any obvious deviations from homoscedasticity or normality. P-values were obtained by likelihood ratio tests (ANOVA) of the full model with the effect in question against the model without the effect in question. Also in this case, the significant results were the same as in the base model unadjusted for medication load described in the main text (p<0.05 for all comparisons).

Finally, to investigate whether specific classes of medications were associated with occurrences of certain μCAP, medication was grouped according to the main neurotransmitter target: GABA, 5HT/DA (serotonin and dopamine antagonists), DA (dopamine partial agonists, e.g. aripiprazole), GABA/5HT/DA (patients using multiple types of medication, or medication with multiple mechanisms of action), specific medications (such as lithium or lamotrigine), and others (e.g. melatonin). Missing data were imputed as NA during the analysis. The effect of this classification of medication was then tested in a LMM of the relationship between μCAPs occurrences and medication class, including sex and age as covariates, by the following R-based LMM formula syntax:

occurrences ~ medication * μCAPs + sex + age, random=~1|subject

where the independent variable “medication” refers to medication classes, “μCAPs” to each μCAP, the fixed factors are sex and age of the subjects. We included a random effect for each subject. Visual inspection of residual plots did not reveal any obvious deviations from homoscedasticity or normality. P-values were obtained by likelihood ratio tests (ANOVA) of the full model with the effect in question against the model without the effect in question and did not show any difference in μCAPs occurrences between each medication class (p>0.05 for all comparisons).

- 1. ***Sub-analyses on motion***

There was no difference in average framewise displacement (FD, a measure of participant’s movement during the fMRI scan) among groups (BD, BDoff, HC), nor among μCAPs (p>0.05 for all comparisons).

- 1. ***Sub-analyses on probability of transition from a μCAPs to the other***

The probability that brain activity transitioned from SMN-μCAPs to SMN-μCAPs was significantly lower in BD patients and BD offspring compared to HC (p<0.0001 for both comparisons). This higher stability of the SMN-μCAPs is coherent with the higher occurrences of SMN-μCAPs in HC than in the other two groups and is thus in agreement with our findings.

There were no significant differences in transition probabilities among the other μCAPs.

- 1. ***Sub-analyses on age***

BD patients were significantly older than the other two groups (p<0.05 for all comparisons). Also for this reason, we included age as a covariate both in the LMM main analyses and in the partial correlations analyses.

- 1. ***Sub-analyses on clinical scores***

MADRS scores were higher in BD patients compared to BDoff p =0.01) and HC (p =0.0002). The addition of MADRS as a confounding variable, besides age and sex, to the base LMM (as follows) did not change the significant results.

occurrences ~ group * μCAPs + sex + age + MADRS, random=~1|subject

However, the fitness of this model (as evaluated through AIC and BIC) decreased compared to the base model (described in the main text). For this reason, we did not include MADRS in the final base model. Additionally, there were no significant differences between groups in the correlation between the ratio between DMN/SMN μCAPs occurrences and MADRS scores (p>0.05).

Non-adaptive CERQ scores modulated the interactions between networks in a similar way as MADRS scores did (**Figure 2**). Indeed, CERQ scores were higher in BD compared with HC (p=0.04), and MADRS and non-adaptive CERQ scores were highly correlated between themselves in the BD groups (rho:0.7; p= 0.001).

Scores of the RRS and of the brooding subscale of the RRS were higher in BD compared with HC (p= 0.031 and p= 0.040, respectively). ALS scores were higher in BD compared with HC (p=0.006). Due to several missing values that could reduce the statistical power of the analysis, we did not compute the aforementioned analyses including other clinical scores such as the mania score (YMRS, see **Table 1**). However, there were no significant differences among groups in the correlations between YMRS and μCAPs occurrences. No differences in other clinical scores between BDoff and the other two groups were identified (p>0.05 for all comparisons).

- 1. ***Non-significant findings on temporal dynamics of hippocampal dFC***

dFC between the hippocampal lateral body and the FPN-μCAP was not different between BD, BDoff, and HC, in agreement with our previous results(18) and the with existing literature(19).

An intriguing finding in our study is the absence of a significant difference in hippocampal medial body dFC with the DMN-μCAP between the three groups. Considering that our overall findings suggest increased self-focused and internal processing in BD and BDoff, one might expect the occurrences of DMN-μCAP to be higher in these groups compared to HC. However, our results are consistent with existing findings from a systematic review on euthymic BD(20), where DMN abnormalities have been predominantly highlighted in unipolar and bipolar depression(21). It's worth noting that our BD patients were clinically euthymic, despite exhibiting sub-threshold depressive and emotion dysregulation symptoms as discussed in the main text. Therefore, it is possible that relatively euthymic BD patients may not exhibit significant DMN dFC abnormalities or that any potential abnormalities are subtle, and our study may not have been powered enough to detect them.

Additionally, as described in the main text, the correlation between the FPN and DMN was significant only in BD patients. This might suggest that there are indeed between-network DMN abnormalities in our sample, but the specific intra-network dFC between the hippocampus and the DMN remains unaffected by the BD diagnosis. These results highlight the complex and nuanced nature of brain connectivity patterns in BD and offer valuable insights into the potential differences in network dynamics between BD patients and HC.

1. **SUPPLEMENTARY DISCUSSION**
   1. ***μCAPs activity-based hippocampal parcellation converges with existing functional and structural segmentations of the hippocampus***

In addition to what has been described in the main text about how the μCAPs activity-based hippocampal parcellation converges with existing functional and structural segmentations of the hippocampus, we add here that the FPN-μCAP was coactive with a data-driven parcellations of the hippocampal lateral body. On the other hand, the DMN-μCAP was coactive with a parcellations of the hippocampal medial body (**Supplementary Table 1**). These functional parcellation of the hippocampal body converge with and expand a previous resting-state FC study that found that the hippocampal body was connected with the DMN and the FPN(22).

1. **SUPPLEMENTARY TABLE**

| **Large-scale brain network** | **Hippocampal subdivisions** | **Significant differences** |
| --- | --- | --- |
| Frontoparietal (FPN) | Lateral Body  (CA1 body) | / |
| Somatomotor (SMN) | Anterior Body  (CA2-4; DG body) | HC>BDoff; HC>BD |
| Limbic (LN) | Head  (Subiculum, DG head) | BD>HC |
| Default Mode (DMN) | Medial Body  (CA4, DG body) | / |
| Salience (SN) | Lateral Tail  (CA2-3, DG tail) | / |

**Supplementary Table 1. Association between large scale networks and hippocampal subdivisions in the μCAPs.** The table shows which large-scale brain network was associated with which specific hippocampal subdivisions. Bipolar disorder (BD), Bipolar Disorder patients’ Offspring (BDoff), Cornus Ammonis (CA), Dentate Gyrus (DG) Healthy Controls (HC).

1. **SUPPLEMENTARY REFERENCES**

1. Harvey PD, Greenberg BR, Serper MR. The affective lability scales: Development, reliability, and validity. J Clin Psychol. 1989;45(5):786–93.

2. Garnefski N, Kraaij V, Spinhoven P. Negative life events, cognitive emotion regulation and emotional problems. Pers Individ Dif. 2001;30(8):1311–27.

3. Treynor W, Gonzalez R, Nolen-Hoeksema S. Ruminative reconsiderd: A psychometric analysis. Cognit Ther Res. 2003;27(3):247–59.

4. Zöller D, Schaer M, Scariati E, Padula MC, Eliez S, Van De Ville D. Disentangling resting-state BOLD variability and PCC functional connectivity in 22q11.2 deletion syndrome. Neuroimage. 2017 Apr 1;149:85–97.

5. Ashburner J, Friston KJ. Unified segmentation. Neuroimage. 2005 Jul 1;26(3):839–51.

6. Ashburner J. A fast diffeomorphic image registration algorithm. Neuroimage. 2007 Oct 15;38(1):95–113.

7. Chao-Gan Y, Yu-Feng Z. DPARSF: A MATLAB toolbox for “pipeline” data analysis of resting-state fMRI. Front Syst Neurosci. 2010;4.

8. Power JD, Barnes KA, Snyder AZ, Schlaggar BL, Petersen SE. Spurious but systematic correlations in functional connectivity MRI networks arise from subject motion. Neuroimage. 2012 Feb 1;59(3):2142–54.

9. Vidaurre D, Smith SM, Woolrich MW. Brain network dynamics are hierarchically organized in time. Proc Natl Acad Sci U S A. 2017;114(48):12827–32.

10. Liu X, Zhang N, Chang C, Duyn JH. Co-activation patterns in resting-state fMRI signals. Neuroimage. 2018;180(February):485–94.

11. Bolton TAW, Tuleasca C, Wotruba D, Rey G, Dhanis H, Gauthier B, et al. TbCAPs: A toolbox for co-activation pattern analysis. Neuroimage. 2020;211:116621.

12. Rey G, Bolton TAW, Gaviria J, Piguet C, Preti MG, Favre S, et al. Dynamics of amygdala connectivity in bipolar disorders: a longitudinal study across mood states. Neuropsychopharmacology. 2021;46(9):1693–701.

13. Bolton TAW, Wotruba D, Buechler R, Theodoridou A, Michels L, Kollias S, et al. Triple Network Model Dynamically Revisited: Lower Salience Network State Switching in Pre-psychosis. Front Physiol. 2020;11(February):1–10.

14. Delavari F, Sandini C, Kojovic N, Saccaro LF, Eliez S, Van De Ville D, et al. Thalamic contributions to psychosis susceptibility: Evidence from co-activation patterns accounting for intra-seed spatial variability (µCAPs). 2023; Available from: https://doi.org/10.1101/2023.05.23.541896

15. Gaviria J, Rey G, Bolton T, Ville D Van De, Vuilleumier P. Dynamic functional brain networks underlying the temporal inertia of negative emotions. Neuroimage. 2021 Oct 15;240.

16. Bolton TAW, Tuleasca C, Wotruba D, Rey G, Dhanis H, Gauthier B, et al. TbCAPs: A toolbox for co-activation pattern analysis. Neuroimage [Internet]. 2020;211:116621. Available from: https://www.sciencedirect.com/science/article/pii/S1053811920301087

17. Kuhn HW. Variants of the hungarian method for assignment problems. Naval Research Logistics Quarterly. 1956 Dec;3(4):253–8.

18. Saccaro LF, Gaviria J, Ville D Van De, Piguet C. Dynamic functional hippocampal markers of residual depressive symptoms in euthymic bipolar disorder. Brain Behav. 2023 Jun 1;13(6).

19. Chou T, Dougherty DD, Nierenberg AA, Deckersbach T. Restoration of default mode network and task positive network anti-correlation associated with mindfulness-based cognitive therapy for bipolar disorder. Psychiatry Res Neuroimaging. 2022;319(July 2021):111419.

20. Syan SK, Smith M, Frey BN, Remtulla R, Kapczinski F, Hall GBC, et al. Resting-state functional connectivity in individuals with bipolar disorder during clinical remission: A systematic review. Journal of Psychiatry and Neuroscience. 2018;43(5):298–316.

21. Piguet C, Karahanoğlu FI, Saccaro LF, Van De Ville D, Vuilleumier P. Mood disorders disrupt the functional dynamics, not spatial organization of brain resting state networks. Neuroimage Clin. 2021;32.

22. Zhong Q, Xu H, Qin J, Zeng LL, Hu D, Shen H. Functional parcellation of the hippocampus from resting-state dynamic functional connectivity. Brain Res. 2019 Jul 15;1715:165–75.
